# Chronic Inflammation Modulates Opioid Receptor Gene Expression and Triggers Respiratory Burst in a Teleost Model

**DOI:** 10.3390/biology11050764

**Published:** 2022-05-17

**Authors:** Diogo Peixoto, Marina Machado, Rita Azeredo, Benjamín Costas

**Affiliations:** 1 ICBAS—Instituto de Ciências Biomédicas Abel Salazar, Universidade do Porto, 4050-313 Porto, Portugal; 2 CIIMAR—Centro Interdisciplinar de Investigação Marinha e Ambiental, 4450-208 Matosinhos, Portugal; mcasimiro@ciimar.up.pt (M.M.); mleme@ciimar.up.pt (R.A.); 3 Departamento de Biología, Facultad de Ciencias del Mar y Ambientales, Instituto Universitario de Investigación Marina (INMAR), Campus de Excelencia Internacional del Mar (CEIMAR), Universidad de Cádiz, 11519 Puerto Real, Spain

**Keywords:** European seabass, chronic inflammation, opioid receptors, immune status

## Abstract

**Simple Summary:**

The natural presence of opportunistic pathogens in aquatic rearing systems in alignment with favorable conditions (compromised fish immune status and/or inappropriate rearing conditions) might result in serious acute disease episodes that can develop into chronic immune responses. The present study characterizes molecular, cellular and humoral markers of chronic inflammation in a fish species with high commercial value. The intense recruitment of immune cells to the inflammatory focus 21 days after triggering an immune response illustrates a clear chronic character. The cellular response was also noticed with circulating leukocyte numbers rising in the blood of the inflamed fish. Furthermore, the cellular-mediated respiratory burst peaked at 21 days post-injection, suggesting that phagocytes were still actively fighting the inflammatory agent. Regarding the molecular analysis, certain genes appear to be good markers of a chronic inflammation response due to their importance in pathways with high relevance in chronic inflammation settings. The present study can serve as a baseline to assess long-term immune-related responses in future studies.

**Abstract:**

Stress-inducing husbandry and rearing conditions, bacterial infections or parasitic diseases may all lead to chronic inflammation. The immune response will then channel energy away from growth, reproduction and other important physiological processes, to fuel immune-related metabolic responses. The present study aims to unravel the mechanisms and contribute with new information on the molecular, cellular and humoral parameters of European seabass (*Dicentrarchus labrax*) undergoing chronic inflammation that can be used as health indicators for application in fish health management. European seabass individuals were intra-peritoneally injected with either Freund’s Incomplete Adjuvant (FIA) to induce inflammation or Hanks Balanced Salt Solution (HBSS) to serve as sham. Fish were sampled at 24 h, 7, 14 and 21 days post-injection and blood, plasma and head-kidney were collected. The results found were clear indicators of an inflamed peritoneal cavity and an ongoing systemic immune response that persisted for at least 21 days. Locally, inflammation was characterized by an intense recruitment of immune cells that was still evident 21 days after injection, thus illustrating the chronic character of the immune response. Cellular response was also noticed peripherally with leukocyte numbers rising in the blood of FIA-injected fish. Furthermore, the cellular-mediated respiratory burst peaked at 21 days post-FIA injection, suggesting that phagocytes were still actively fighting the phlogistic agent. Regarding the head-kidney molecular analysis, *cxcr4* and *il34* appear to be good markers of a chronic inflammation response due to their importance for pathways with high relevance in chronic inflammation settings. In addition, opioid receptor *nopr* seems to be a good marker of a chronic inflammation response due to its role in detecting noxious stimuli. The present study can serve as a baseline to assess long-term immune-related responses in future studies. For that, more research is nonetheless required to select more responsive and specific molecular markers.

## 1. Introduction

Increasing consumer concerns about the quality, safety, freshness and health value aquaculture products have been pushing farmers into becoming increasingly aware of fish welfare and on how intimately connected it is with final product quality [1,2]. Fish health is compromised by several external and internal factors that often occur in a persistent manner throughout the production cycle. The natural presence of opportunistic pathogens in rearing systems in alignment with favorable conditions (compromised fish immune status and/or inappropriate rearing conditions) might result in serious acute disease episodes, or even develop into a chronic immune response. Chronic immune responses might also arise with the misuse of alternative ingredients in aquafeeds, given the high amount of antinutritional factors and/or due to an inadequate feed formulation/regime [3]. On top of that, these situations might be exacerbated by rearing conditions such as high animal densities. It is now generally accepted that stressful rearing conditions are described as affecting flesh quality (lower muscle pH and faster meat quality deterioration), while decreasing growth rates and rendering fish more prone to pathologies [1,4,5,6,7,8,9,10].

When antigens are detected by the host immune recognition complexes, an array of soluble and cellular defense mechanisms is activated, sustained by both the head-kidney and blood, to fuel a local inflammatory response. Inflammation is a protective reaction of the host in response to injury, infection, or simply the presence of a foreign body, that consists of complex series of homeostatic mechanisms affecting the immune, nervous and circulatory systems [11,12,13]. Inflammatory processes are orchestrated by pro- and anti-inflammatory mediators working in different timeframes and aimed at eliminating the threat and achieving a new homeostasis without or with limited self-damage [14]. Despite its protective character, it involves an increase in the oxidative stress with increased production of reactive oxygen and nitrogen species that are not only deleterious for the invading microorganism but also for the host. Even more so, an unrestrained inflammatory response is often linked to an extended exhaustion of resources, tissue damage and fibrosis [15,16,17].

Fish opioid receptors have been shown to be expressed in the hypothalamus, pituitary gland and head-kidney with a huge range of physiological roles, including nociception and cognition, as well as affective, endocrine, cardiovascular, gastrointestinal, immune, metabolic and respiratory functions, stress and autonomic regulation [10,18]. A vice versa modulation occurs between the immune and neuroendocrine systems where they both share a network of compounds and their receptors that are involved in the regulation of both responses. As active compounds of this network, opioids and their receptors participate in regulatory mechanisms of the cellular immune response, as demonstrated by the immunomodulatory role of Mu opioid (*mu*) on immune cell migration [17] and by regulatory functions on neuroendocrine and immune responses in common carp, *Cyprinus carpio* L. [19].

A prolonged inflammation involves longer time periods in which more energy and resources are allocated to cope with the demands of the response [9]. At this point, it may develop into a systemic situation typically associated with leukocytosis, activation of complement cascades, lowering plasma iron levels and amino acid mobilization from muscle to liver for intense protein synthesis and secretion [14,20]. Chronic immune responses are, therefore, highly energy and nutrient demanding, thus deviating resources away from other key physiological processes such as growth and reproduction.

Despite a reasonable amount of studies on fish immune responses, there is no solid chronic inflammation model to be applied in teleost fish studies and a battery of biological markers that can more accurately characterize this particular immune context. The present study aims to gather information on the molecular, cellular and humoral parameters of European seabass (*Dicentrarchus labrax*) undergoing chronic inflammation that can be used as health indicators for application in fish health management.

## 2. Materials and Methods

### 2.1. Experimental Design

**Figure 1:**
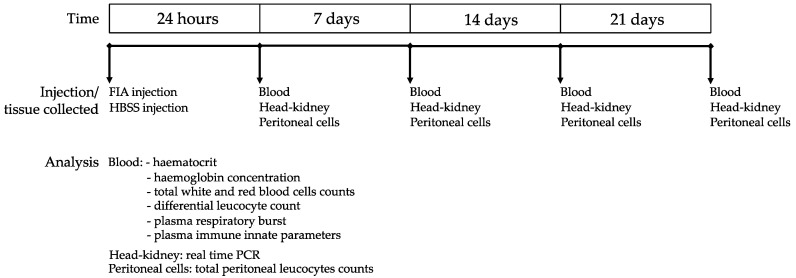
Experimental design.

European seabass were acquired from a certificated hatchery (MARESA, Ayamonte, Spain) and maintained in quarantine for 2 weeks at the Centro Interdisciplinar de Investigação Marinha e Ambiental (CIIMAR; University of Porto, Portugal) fish holding facilities under culture conditions described below. Groups of 24 individuals (≈300 g) were randomly distributed into 2 tanks (3000 L) of a recirculating seawater system in which O^2^ saturation (7.38 ± 0.01 mg L^−1^), salinity (35), temperature 20 ± 0.5 °C and photoperiod (10:14 h dark:light) were kept unchanged throughout the experiment. Ammonium and nitrite levels were kept below 0.025 and 0.3 mg L^−1^, respectively. At the beginning of the trial, 100 μL of Freund’s Incomplete Adjuvant (FIA; Sigma-Aldrich, St. Louis, MI, USA) were intra-peritoneally (i.p.) injected in half of the fish (FIA group) to induce inflammation, while the other half was injected with 100 μL of a saline solution (Hanks’ Balanced Salt solution; HBSS, Sigma-Aldrich) to serve as sham (CTRL group). The trial lasted for 21 days and during this period the individuals were daily hand-fed a commercial diet. At 24 h, 7, 14 and 21 days after i.p. injection, 6 fish from each tank were euthanized by an overdose of anesthetic (2-phenoxyethanol) and weighed. Blood was sampled from the caudal vein using heparinized syringes and fresh samples were used to assess the hematological profile and extracellular respiratory burst. The remaining blood was centrifuged at 10,000× *g* for 10 min at 4 °C, and plasma was collected and stored at −80 °C for evaluating innate humoral immune response parameters. Peritoneal exudates were collected afterwards as described below, and the head-kidney was sampled and stored at −80 °C until further processing for gene expression studies (Figure 1). No mortality was observed during the trial. Experiment trials were directed by trained scientists (following FELASA category C recommendations) and conducted according to the guidelines on the protection of animals used for scientific purposes from the European directive 2010/63/UE.

### 2.2. Peritoneal Exudates

Peritoneal cells were collected according to the procedure described by [21,22]. Quickly, 5 mL of cold HBSS supplemented with 30 units heparin mL^−1^ was injected into the peritoneal cavity. The peritoneal area was then slightly massaged and the exudate containing suspended cells was withdrawn. Finally, total peritoneal leukocytes counts were performed using a hemocytometer.

### 2.3. Hematological Parameters

The hematological profiling was performed according to Machado et al. [23] and comprised the analysis of hematocrit, hemoglobin concentration, total white (WBC) and red (RBC) blood cells counts as well as differential leukocyte counts. Immediately after blood collection, blood smears were performed and air dried. Fixation and staining protocols (Wright’s stain; Hemacolor) were performed according to Afonso et al. [24] including peroxidase granule staining step in order to facilitate neutrophil detection. Slides were examined under optical microscope (×1000) and at least 200 leukocytes per slide were counted and classified as thrombocytes, lymphocytes, monocytes and neutrophils. Absolute concentration of each leukocyte type was calculated based on total WBC counts (×10^4^ mL^−1^).

### 2.4. Respiratory Burst

Respiratory burst in peripheral leukocytes was evaluated according to Nikoskelainen et al. [25] with some modifications [21]. Briefly, 4 μL of blood was added to 96 μL of HBSS in a 96-well flat bottom (white plate). Then, 100 μL of freshly prepared luminol solution (2 mM luminol in 0.2 M borate buffer pH 9.0, with 2 μg mL^−1^ phorbol 12-myristate 13-acetate) was added to each well. Luminol-amplified chemiluminescence was measured every 3 min for 2 h in a luminescence reader for generation of kinetic curves. The integral luminescence in relative light units was calculated.

### 2.5. Plasma Innate Immune Parameters

The following parameters were previously validated in plasma samples of European seabass. All analyses were conducted in triplicate and absorbance was read on a microplate reader.

#### 2.5.1. Antiprotease Activity

Antiprotease activity was determined as described by Ellis [26]. Briefly, 10 μL of plasma was incubated with 10 μL of trypsin solution (5 mg mL^−1^ in sodium bicarbonate (NaHCO_3_), 5 mg mL^−1^, pH 8.3) for 10 min at 22 °C in polystyrene microtubes. Then, 100 μL of phosphate buffered saline (NaH_2_PO_4_, 13.9 mg mL^−1^, pH 7.0) and 125 μL of azocasein (20 mg mL^−1^ in NaHCO_3_, 5 mg mL^−1^, pH 8.3) were added and the mixture was incubated for 1 h at 22 °C. Finally, 250 μL of trichloroacetic acid (TCA) was added to each microtube and incubated for 30 min at 22 °C. The mixture was centrifuged at 10,000× *g* for 5 min at room temperature. Afterwards, 100 μL of the supernatant was transferred to a 96-well plate that contained 100 μL of NaOH (40 mg mL^−1^) per well. The absorbance was read at 450 nm. Phosphate buffer in place of plasma and trypsin served as blank, whereas a reference sample was prepared using phosphate buffer in place of plasma samples. The percentage inhibition of trypsin activity compared to the reference sample was calculated.

#### 2.5.2. Protease Activity

Protease activity was quantified using the azocasein hydrolysis assay according to Azeredo [14], using 10 μL of plasma sample and adding 100 μL of NaH_2_PO_4_ (13.9 mg mL^−1^, pH 7.0) and 125 μL azocasein (20 mg mL^−1^ in NaHCO_3_, pH 8.3). After incubation for 24 h at 22 °C in polystyrene microtubes, 250 μL of 10% TCA was added to each mixture, followed by centrifugation (10,000× *g* for 5 min). In a microplate, 100 μL of 1 N NaOH was added to 100 μL of the supernatant and the absorbance was read at 450 nm. The percentage of protease activity compared to the reference sample (trypsin solution, 5 mg mL^−1^ in NaHCO_3_, pH 8.3) was calculated.

#### 2.5.3. Lysozyme Concentration

Lysozyme concentration was measured using a turbidimetric assay as described by Ellis [27]. Briefly, a solution of *Micrococcus lysodeikticus* (0.5 mg mL^−1^, 0.05 M sodium phosphate buffer, pH 6.2) was prepared and added (250 μL) to 10 μL of each plasma sample in a microplate to give a final volume of 260 μL. The reaction was carried out at 25 °C and the absorbance was measured at 450 nm after 0.5 and 5 min. Lyophilized hen’s egg white lysozyme was diluted in sodium phosphate buffer (0.05 M, pH 6.2) and used to develop a standard curve from which the amount of lysozyme in each sample was calculated.

#### 2.5.4. Peroxidase Activity

Total peroxidase activity in plasma was measured according to Quade [28]. Briefly, 15 μL of each sample was diluted in 135 μL of HBSS without Ca^+2^ and Mg^+2^ in flat-bottomed 96-well plates. Then, 50 μL of 20 mM 3,3′,5,5′-tetramethylbenzidine hydrochloride (TMB) and 50 μL of 5 mM hydrogen peroxide (H_2_O_2_) were added. The color-change reaction was stopped after 2 min by adding 50 μL of 2 M sulfuric acid (H_2_SO_4_) and the OD was read at 450 nm. HBSS instead of plasma was used to serve as blank. One unit of peroxidase activity (units mL^−1^ sample) was defined by the quantity of peroxidase that produces an absorbance change of 1 OD.

#### 2.5.5. Immunoglobulin M

Plasma immunoglobulin M (IgMp) levels were analyzed by an enzyme-linked immunosorbent assay (ELISA) [29,30]. Plate wells were coated with plasma proteins, washed 3 times with T-TBS (1× Tris-buffered saline and 0.1% Tween 20, pH 7.3), blocked for 2 h at room temperature with blocking buffer (5% low fat milk in T-TBS) and rinsed again with T-TBS. Plates were then incubated for 1 h with 100 μL per well of anti-European seabass IgM monoclonal antibody (1:100 in blocking buffer), washed and incubated with the secondary antibody anti-mouse IgG-HRP (1:1000 in blocking buffer). Afterwards, 100 μL of TMB was added and the color-change reaction was stopped after 5 min by adding 100 μL of 2 M H_2_SO_4_. The OD was read at 450 nm. Negative controls consisted of samples without plasma or without primary antibody, from which OD values were subtracted for each sample.

### 2.6. Gene Expression Analysis

Head-kidney samples were used for total RNA isolation using the NZY Total RNA Isolation kit (NZYTech) following manufacturer’s instructions and first-strand cDNA was synthesized with NZY First-Strand cDNA Synthesis Kit (NZYTech). DNA amplification was carried out with specific primers for each gene that had been selected for its involvement in immune responses and oxidative stress. Sequences encoding European seabass *il34*, *ptx*, *tgfβ*, *cxcr4*, *il1β*, *casp1*, *il10*, *inf-y*, *IgM*, *mmp9*, *muor*, *kor1*, *nopr*, *dor2* and *ogfr1* were identified after carrying out a search in the databases and primers were designed as described in [31]. Accession number, efficiency values, annealing temperature, product length and primers sequences are presented in Table 1. Real-time quantitative PCR was carried out in a CFX384 Touch Real-Time PCR Detection System (Biorad), using 4.4 μL of diluted cDNA mixed with 5 μL of iTaq Universal SYBR Green Supermix (BioRad) and 0.3 μL (10 μM) of each specific primer in a final volume of 10 μL. The standard cycling conditions were 95 °C initial denaturation for 10 min, followed by 40 cycles of two steps (95 °C denaturation for 15 s followed by primer annealing temperature for 1 min), 95 °C for 1 min followed by 35 s at the annealing temperature, and finally, 95 °C for 15 s. All reactions were carried out as technical duplicates. Melting curve analysis was also performed to verify that no primer dimers were amplified. The expression of the target genes was normalized using the expression of European seabass elongation factor 1-α (*ef1α*), 40s ribosomal protein (*40s*) and ribosomal protein L13 mRNA (*i13α*).

### 2.7. Data Analysis

All results are expressed as mean ± standard deviation (SD). Shapiro–Wilk test was used for normality of variances, as well as Pearson skewness coefficient. When normality was not observed, a non-parametric test with Kruskal–Wallis pairwise comparisons was used to compare significant differences in all the parameters. Differences were tested by a two-way ANOVA, with time and treatment (FIA or CTRL) as factors, followed by a *post-hoc* Tukey HSD test, used to identify significant differences amongst groups. Statistical analyses were carried out using IBM SPSS v27.0 Statistics with a significance level of 95% (*p* ≤ 0.05). A principal component analysis (PCA) using Addinsoft XLSTAT 2021 system software and a discriminant analysis (DA) using IBM SPSS v27.0 Statistics were applied to assess the consensus among the variables that differed significantly among dietary treatments.

## 3. Results

### 3.1. Hematological Response

**Figure 2:**
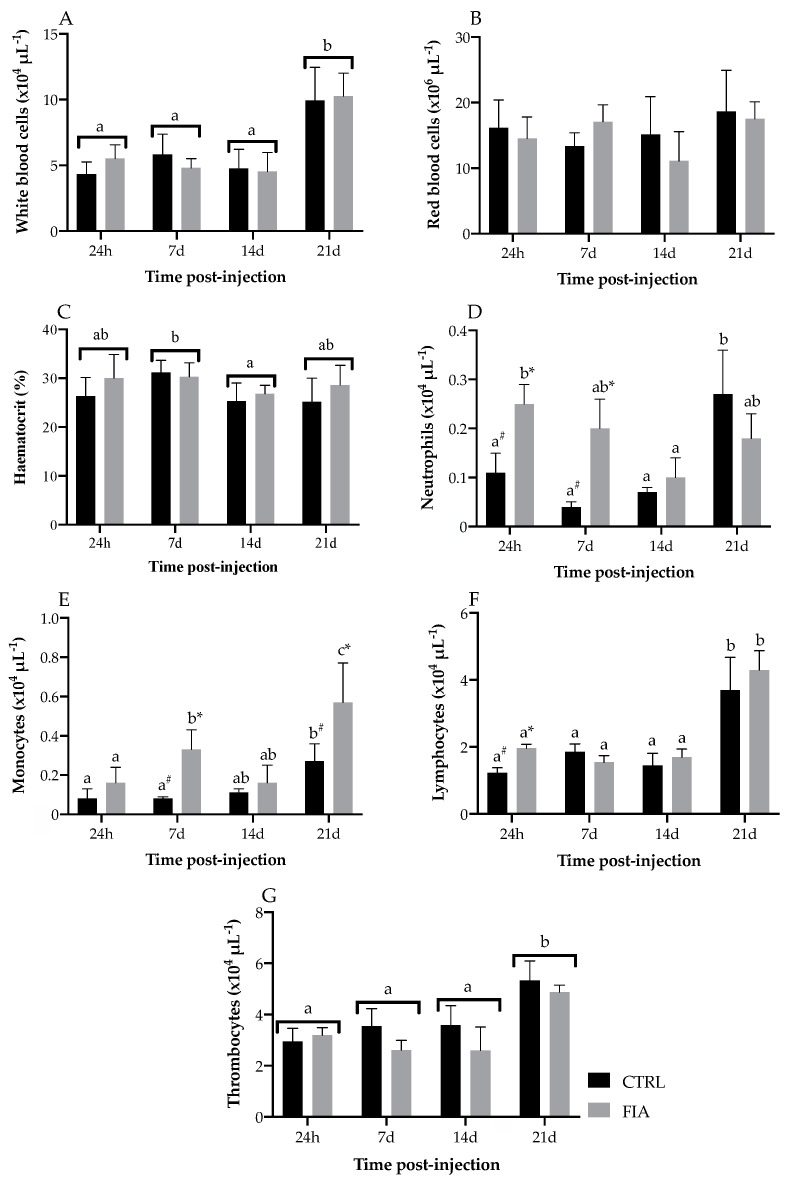
Absolute values of total white and red, peripheral leukocytes (neutrophils, monocytes, lymphocytes and thrombocytes) of European seabass sampled at 24 h, 7, 14 and 21 days after i.p. injection. (**A**)—white blood cells, (**B**)—red blood cells, (**C**)—hematocrit, (**D**)—neutrophils, (**E**)—monocytes, (**F**)—lymphocytes, (**G**)—thrombocytes. Values are presented as means ± SD (*n* = 6). Different low case letters stand for statistically significant differences attributed to sampling time. Symbols stand for significant differences between stimuli. (Two-way ANOVA; Tukey *post-hoc* test; *p* ≤ 0.05).

The complete set of results is available in the Supplementary File (Table S1). The total white blood cell population increased at 21 days post-injection regardless of treatment, while no significant differences were observed in the total red blood cell population (Figure 2A,B). The hematocrit decreased from 7 to 14 days post-injection regardless of treatment but levels were observed to increase back to values similar to those found in the first sampling time (Figure 2C). Peripheral neutrophils were higher in FIA-injected fish than in the CTRL group in the first two sampling points and concentration decreased from 24 h to 14 days post-injection in inflamed fish (Figure 2D). In contrast, in the CTRL group, neutrophils were higher at 21 days compared to all other sampling points. Monocyte concentration was higher in FIA-injected fish compared to the levels in the CTRL group at 7 and 21 days post-injection and it significantly increased over time in inflamed fish (Figure 2E). Monocytes were also observed to increase in time in FIA-injected fish, peaking at 21 days post-injection. FIA-injected fish had higher lymphocyte numbers at 24 h post-injection compared to their CTRL counterparts, but a significant increase was observed in both groups at 21 days, relative to the other sampling points (Figure 2F). Peripheral thrombocyte concentration was observed to increase at 21 days post-injection relative to the other sampling points regardless of treatment, while CTRL fish had a higher concentration than FIA-injected fish, regardless of time of sampling (Figure 2G).

### 3.2. Total Peritoneal Cell Counts and Respiratory Burst of Circulating Leukocytes

**Figure 3:**
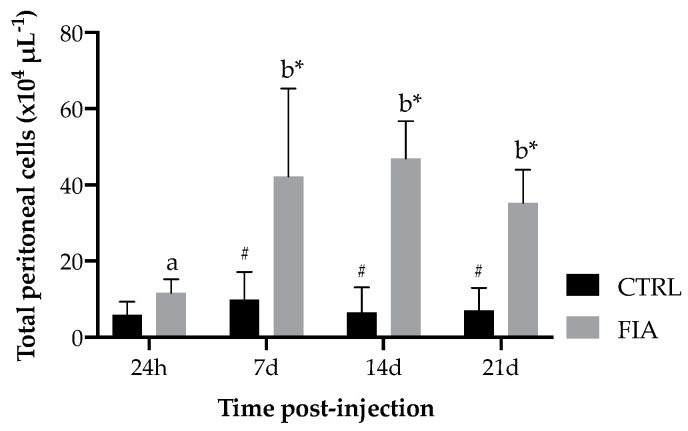
Absolute values of total peritoneal leukocytes of European seabass sampled at 24 h, 7, 14 and 21 days after i.p. injection. Values are presented as means ± SD (*n* = 6). Different low case letters stand for statistically significant differences attributed to sampling time. Symbols stand for significant differences between stimuli. (Two-way ANOVA; Tukey *post-hoc* test; *p* ≤ 0.05).

Regarding total peritoneal cells, an increase was observed in FIA-injected fish sampled at 7 days after i.p. injection compared to those sampled at 24 h. Leukocyte numbers remained high in this group until the last sampling point. Moreover, total peritoneal cell numbers in FIA-injected fish were significantly higher than those of CTRL fish at 7, 14 and 21 days post-injection (Figure 3).

**Figure 4:**
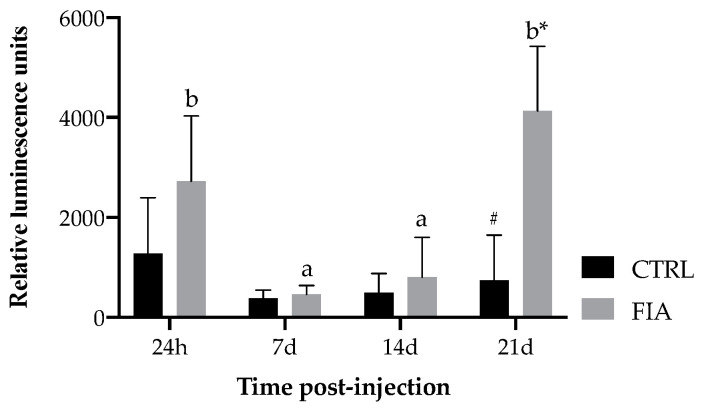
Relative luminescence units of respiratory burst of European seabass sampled at 24 h, 7, 14 and 21 days after i.p. injection. Values are presented as means ± SD (*n* = 6). Different low case letters stand for statistically significant differences attributed to sampling time. Symbols stand for significant differences between stimuli. (Two-way ANOVA; Tukey *post-hoc* test; *p* ≤ 0.05).

Respiratory burst decreased from 24 h to 7 days post-injection in FIA-injected fish, and remained low until it increased again from 14 to 21 days following FIA injection (Figure 4).

### 3.3. Innate Humoral Parameters

The complete set of results is available in the Supplementary File (Table S2).

**Figure 5:**
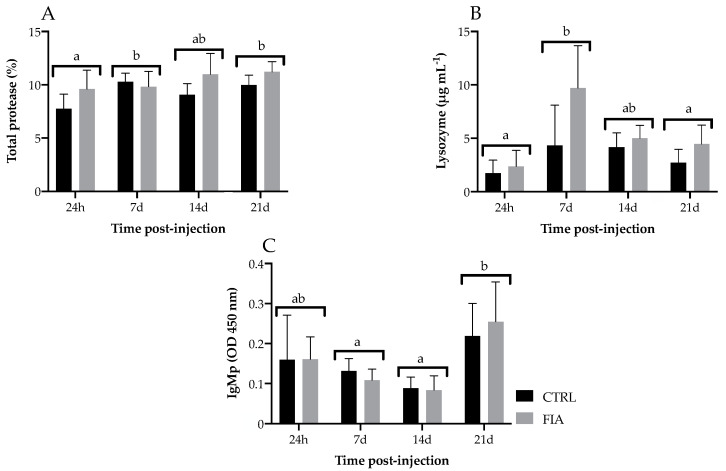
Plasma innate immune response parameters of European seabass sampled at 24 h, 7, 14 and 21 days after i.p. injection. (**A**)—total protease content, (**B**)—lysozyme and (**C**)—immunoglobulins M. Values are presented as means ± SD (*n* = 6). If interaction was significant, Tukey *post-hoc* test was used to identify differences among treatments. Different low case letters stand for statistically significant differences between sampling points. (Two-way ANOVA; Tukey *post-hoc* test; *p* ≤ 0.05).

Total proteases and lysozyme concentrations were enhanced in FIA-injected fish compared to the CTRL group, regardless of sampling time (Figure 5A,B, respectively). Moreover, total protease activity increased from 24 h to 7 days post-injection regardless of treatment and remained so until the end of the experiment (Figure 5A). Differently, the lysozyme concentration increased at 7 days post-injection regardless of treatment but decreased at 21 days back to levels similar to those observed in the first sampling time (Figure 5B). Plasma IgM remained stable until it peaked at 21 days post-injection, irrespective of treatment (Figure 5C). No significant differences were observed regarding antiprotease and peroxidase activities (Supplementary File, Table S2).

### 3.4. Head-Kidney Gene Expression

The complete set of results is available in the Supplementary File (Table S3).

**Figure 6:**
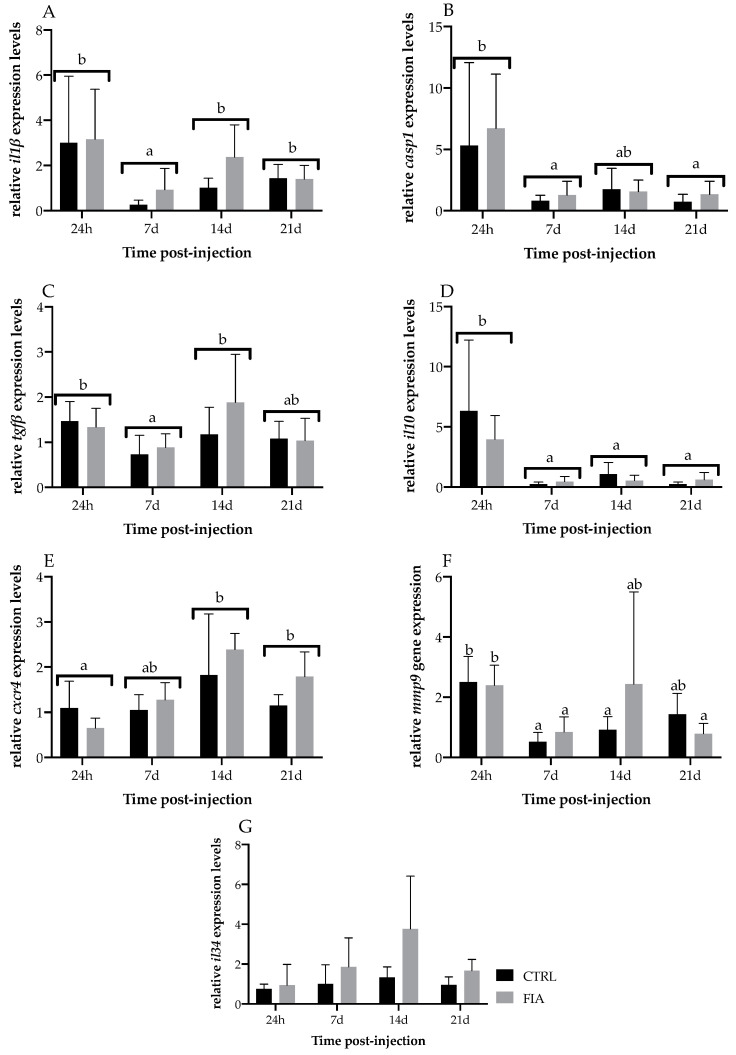
Relative expression of immune-related genes in the head-kidney of European seabass sampled at 24 h, 7, 14 and 21 days after i.p. injection. (**A**)—*il1β*, (**B**)—*casp1*, (**C**)—*tgfβ*, (**D**)—*il10*, (**E**)—*cxcr4*, (**F**)—*mmp9* and (**G**)—*il34*. Values are presented as means ± SD (*n* = 6). If interaction was significant, Tukey *post-hoc* test was used to identify differences among treatments. Different low case letters stand for statistically significant differences between sampling points. (Two-way ANOVA; Tukey *post-hoc* test; *p* ≤ 0.05).

**Figure 7:**
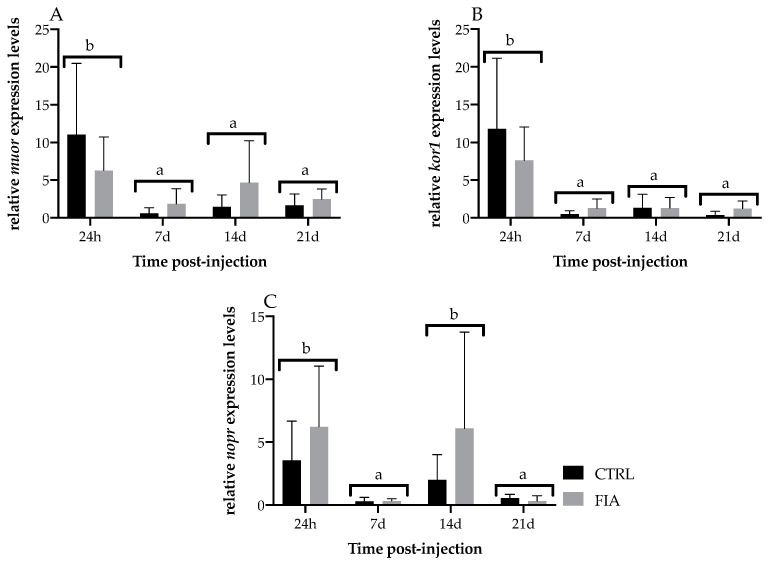
Relative expression of opioid receptor-related genes in the head-kidney of European seabass sampled at 24 h, 7, 14 and 21 days after i.p. injection. (**A**)—*muor*, (**B**)—*kor1* and (**C**)—*nopr*. Values are presented as means ± SD (*n* = 6). If interaction was significant, Tukey *post-hoc* test was used to identify differences among treatments. Different low case letters stand for statistically significant differences between sampling points. (Two-way ANOVA; Tukey *post-hoc* test; *p* ≤ 0.05).

Regarding head-kidney gene expression, *il1β* and *casp1* were both upregulated in FIA-injected fish, irrespective of sampling time (Figure 6A,B, respectively). Moreover *il1β*, *casp1*, *tgfβ* and *il10* relative expressions decreased from 24 h to 7 days regardless of treatment (Figure 6A, B, C and D, respectively). However, while the *tgfβ* and *il1β* decrease was followed by an upregulation at 14 days, *casp1* and *il10* expression remained at lower levels until the end of the experiment. Differently, *cxcr4* relative expression was upregulated at 14 days post-injection, and levels were kept high until the last sampling point, regardless of treatment (Figure 6E). *mmp9* relative expression in FIA-injected and CTRL fish dropped from 24 h to 7 days post-injection and was still lower at 21 days (Figure 6F). Although not statistically significant, *il34* increased in time until 14 days in FIA-injected fish (Figure 6G). Opioid receptors did not significantly differ between FIA and HBSS groups but mu and kappa opioid receptors were downregulated in fish sampled at 7-, 14- and 21-days post-injection compared to levels measured at 24 h (*muor* and *kor1*, Figure 7A and B, respectively). Likewise, the nociception opioid receptor (*nopr*) expression was downregulated from 24 h to 7 days post-injection and levels were still comparably lower at 21 days, irrespective of treatment (Figure 7C). However, *nopr* expression was increased from 7 days to 14 days, regardless of treatment. Pentraxin (*ptx*), immunoglobulin M (*igm*), interferon-γ (*inf-γ*), delta opioid receptor 2 (*dor2*) and opioid growth factor receptor 1 (*ogfr1*) were not significantly modulated by sampling time nor treatment (Supplementary File, Table S3).

**Figure 8:**
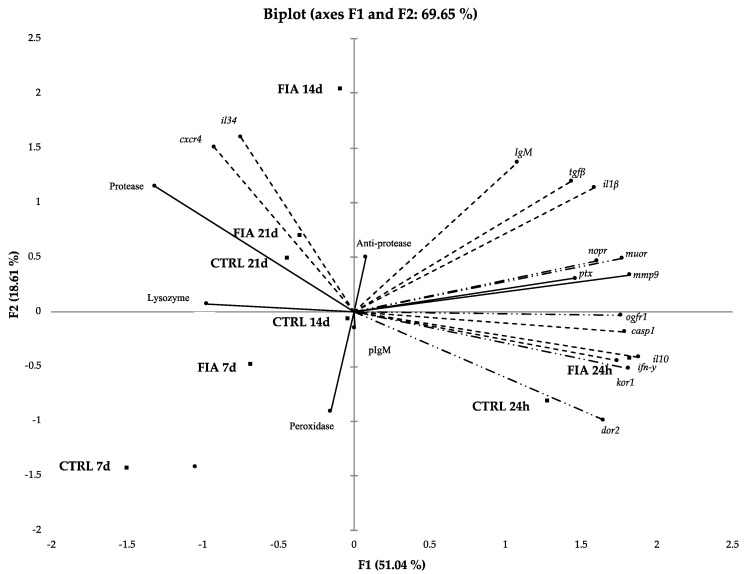
Principal component analysis biplot of the mean scores and loadings for plasma immune and gene expression variables during the inflammation period in European seabass. (—) Plasma immune response, (- - -) gene expression and (—··—) opioid receptors variables at 24 h, 7, 14 and 21 days after FIA- and HBSS-injection. IgMp, plasma immunoglobulins M; *cxcr4*, chemokine CXC receptor 4; *il34*, interleukin 34; *ptx*, C-reactive protein; *igm*, immunoglobulin M; *il1β*, interleukin 1 β; *tgfβ*, transforming growth factor-β; *mmp9*, matrix-metalloproteinase 9; *casp1*, caspase 1; *il10*, interleukin 10; *ifn-γ*, interferon γ; *kor1*, κ-type opioid receptor-like 1; *dor2*, δ-opioid receptor; *nopr*, nociceptin opioid receptor; *muor*, μ opioid receptor and *ogfr1*, opioid growth factor receptor-like protein 1-like.

The principal component analysis (PCA) methodology attempted to identify underlying variables or factors that might explain the pattern of correlations within a set of observed variables. Figure 8 shows the two first dimensions of the PCA consensus, which together account for 69.65% of the variability of the experimental data (F1 51.04%; F2 18.61%), being the overall performance of the analysis medium (Kaiser–Meyer–Olkin index = 0.701; Bartlett’s sphericity test *p* < 0.001). PCA indicated that the fish sampled at 24 h post-injection had different contributions to the model in F1, where FIA-injected fish contributed with 41.5% whereas HBSS-injected fish (CTRL) contributed with 20.6% (see Supplementary File, Tables S4 and S5). Moreover, increased gene expression seemed to be strongly discriminating the group of fish sampled at 24 h post-injection from those sampled at 7, 14 and 21 days post-injection. On the other hand, the humoral parameters were enhanced in these later-sampled groups, except for FIA-injected fish sampled at 21 days which were strongly loaded by *cxcr4* and *il34* gene expression. The discriminant analysis (M Box test *p* < 0.05; Wilk’s Lambda value < 0.2) confirmed a clear separation between FIA- and HBSS-injected fish sampled at 24 h and the other sampling points represented in the biplot in Figure 8, whereas no significant differences among both FIA- and HBSS-injected fish at 7, 14 and 21 days were observed (F1; Figure 8).

## 4. Discussion

Although there are a reasonable number of studies regarding fish immune responses against different stimuli, few are clear about the mechanisms involved in chronic inflammatory insults. Thus, this study aimed to gather information on the molecular, cellular and humoral parameters of European seabass experiencing chronic inflammation which could be used as health indicators for application in fish health management.

Phagocytes are differentiated myeloid cells (mostly neutrophils and macrophages) with a crucial importance in fish innate immune responses, in particular during the inflammatory response, as central regulators of both proinflammatory and homeostatic anti-inflammatory processes [32]. Vasodilatation and cell migration are two classical aspects of the acute phase of inflammation [13] and neutrophils are known to be the first leukocytes recruited to the inflammation site, and to launch an innate immune response [33,34,35]. Accordingly, in this study, peripheral neutrophils were indeed higher in those fish undergoing an inflammatory response and sampled at earlier timepoints (24 h or even 7 days post-injection). At later sampling points, however, neutrophils were no longer standing out in this group, in comparison to the CTRL group. Differently, a clear prolonged cellular response denoting a chronic immune response was displayed by monocytes. Higher peripheral monocyte concentration was observed in FIA-injected fish at 7 days and, as the response progressed in time, it was once higher 21 days after the insult. The absence of differences between FIA and CTRL fish in peripheral monocyte numbers at 14 days post-injection could be related to a slowdown of cell recruitment, since total peritoneal leukocyte concentration in FIA treated fish was already much higher at this timepoint than in CTRL fish. A clear emphasized cell response was observed at the inflammatory focus of FIA-injected fish, as attested by a conspicuous accumulation of leukocytes in the peritoneal cavity 7 days following injection. The numbers were as high at 21 days, suggesting that the inflammatory response was still on course. Similar phagocyte responses were observed in European seabass and Senegalese sole (*Solea senegalensis*) upon i.p. injection of live or inactivated *Photobacterium damselae piscicida*, respectively, with a reduction in peripheral lymphocyte and monocyte numbers, in contrast to an increase in the inflamed peritoneal cavity [23,31,36]. In the present study, the sustained cell recruitment after so many days post-injection (i.e., 21 days) highlights that leukocyte concentration is a good peripheral marker of an activated immune response following chronic immune stimulation in the European seabass.

Fish activated thrombocytes, with regard to their hemostatic and immunological activity, are equivalent to mammalian platelets, taking their part in the process of inflammation following the hemostatic process and thrombus formation [37]. To a certain extent, thrombocytes are also able to perform phagocytosis and have the ability to neutralize and degrade microorganisms accelerating monocytes and dendritic cell activation and antigen presentation to T-cells [37,38,39]. Circulating thrombocyte populations proved to be lower in FIA-injected fish than in the CTRL group. This result is in accordance with previous studies stating that thrombocytes accumulate locally at the damage site, releasing and expressing proteins and substances to deal with the inflammatory insult [37,40,41].

In immune cells performing phagocytosis, this phenomenon is correlated with an increase in oxygen consumption, known as respiratory burst, that is immediately converted into reactive oxygen species [42,43,44,45]. The initial drop observed from 24 h to 7 days post-injection in fish injected with FIA emphasizes the importance of this cytotoxic mechanism in the acute phase of the immune response. Interestingly, this cellular-mediated process peaked at 21 days post-FIA injection suggesting that phagocytes were still actively fighting the phlogistic agent. Since monocytes were also abundant at this time point in the same group, it is likely that these cells were the main players. Respiratory burst is a particularly interesting parameter to be enhanced at such a late time-point, as it is a key discriminant feature of active phagocytes [43], very much present at the onset of inflammation.

Being amongst the most significant innate immune mechanisms, lysozyme activity has an important role against Gram-positive and -negative bacteria, as well as in activating the complement system and phagocytes [46]. Proteases, besides other regulatory mechanisms [47], are also directly involved with the immune response as they cleave the pathogens’ proteins, activate and enhance the production of other immune components such as complement or immunoglobins [48]. Results from the present study point out a clear difference between FIA and CTRL fish, with the fish undergoing an inflammatory response having higher plasma protease activity and lysozyme concentration than those of the CTRL group, regardless of the stage of the response. Yet, as no interactive effects have been observed between inflammation and sampling time in any of the evaluated humoral parameters, their specific behavior throughout the immune response was not distinguishable in the present experiment, despite their importance throughout an activated immune response.

As the inflammatory response continues, there is an intensification of transcriptional activation of several immune-related molecular markers, such as inflammatory cytokines and chemokines that activate and regulate the immune response, antimicrobial peptides, acute phase proteins, and immune stimuli-dependent enzymes, among others [49]. In this study, a principal component analysis (PCA) showed that at 24 h post-infection in both FIA- and HBSS-injected fish, the expression of most genes analyzed in the head-kidney was increased compared to the other sampling points. Moreover, in spite of being not statistically significant, this general molecular upregulation was more prominent in FIA-injected fish than in those from the CTRL group. IL1β is a potent proinflammatory cytokine which induces gene expression of other cytokines such as tumor necrosis factor α, interleukins 1α, 6 and 8, prostaglandin-endoperoxide synthase 2 and monocyte chemoattractant protein 1 [50,51,52]. For full functionality, IL1β has to be cleaved by the cysteine protease caspase 1, which thereby has a regulatory role in the inflammatory response [50,51,52]. Similarly, to that reported in previous studies [53], the highest expression levels of both *il1β* and *casp1* were observed in fish sampled at 24 h, and levels were higher in the FIA-injected compared to the CTRL fish, regardless of sampling point. However, after a first decrease to much lower levels from 24 h to 7 days, head-kidney *il1β* was upregulated again at 14 days, pointing to the ongoing chronic inflammatory response that is not only focused at the inflammation site, but is also triggering a systemic response. The presence of similar gene expression patterns (despite at lower scales) in the CTRL group might be an effect of the injection per se and the associated stress due to handling/air exposure procedures which are known to induce neuroendocrine and immune responses [10].

The anti-inflammatory cytokine TGFβ is a potent negative regulator of hematopoiesis, modulating the proliferation, differentiation and function of several cell types [54]. TGFβ counteracts other earlier cytokine actions (such as IL1β) as the immune response develops, controlling the inflammatory process in a later stage [14,55]. Similarly, IL10 is also a regulatory cytokine described to peak during the late phase of an inflammatory response, which inhibits excessive activation of the immune response and initiate processes of wound healing, tissue remodeling and recovery [10,56,57]. Both genes did not seem to be modulated by the inflammatory stimulus, although *il10* expression significantly dropped from 24 h to 7 days post-injection, regardless of the nature of the injection. Despite these being among the most significant anti-inflammatory mediators, those anti-inflammatory cytokines do not exclusively regulate immune responses. In line with this, the multivariate analysis showed that *cxcr4* and *il34* were particularly important in FIA-injected fish at 14 and 21 days, contrasting with most of the molecular markers discussed previously. Indeed, relatively high levels of *il34* expression across different tissues suggest a regulatory and homeostatic role of *il34* during the immune response [58,59]. It has been reported that IL34 plays a role in macrophage biology and in autoimmune and chronic inflammatory diseases [60]. *cxcr4* is an exclusive receptor of CXC chemokine ligand 12 (*cxcl12*), showing higher expression in immune tissues of teleost fishes [61,62] and functioning to maintain active neutrophils at the inflammatory site [63]. In accordance with results from the present study, van der Sar et al. [63] reported that the *cxcl12*/*cxcr4* signaling axis could modulate the inflammation resolution in zebrafish larvae.

As a compound that, amongst other functions, is involved in leukocyte migration [64], and based on previous studies [53], *mmp9* mRNA expression level was found higher at 24 h post-injection than at subsequent sampling points [65]. Unfortunately, and possibly due to a stress-induced masking effect, no differences were observed between FIA and CTRL groups. However, although not statistically significant, the expression levels of *mmp9* in FIA-injected fish at 14 days post-injection seemed to be higher than those of the CTRL group, suggesting that at this later stage, and in this particular context, chronic inflammation is also marked by a second wave of peripheral cell migration to the inflammation site.

Amongst the broad spectrum of physiological roles that opioids and their receptors seem to play, this study intended to evaluate their ability to regulate the fish immune response. The clear inhibitory effects of morphine—an opiate that binds MUOR—have been observed on the gene expression of head-kidney proinflammatory cytokines and their receptors during the innate immune response of carp, *Cyprinus carpio* [19]. Opioids are also known to be involved in neuroendocrine mechanisms, in which the head-kidney plays a part, too. This multidisciplinary profile of opioids’ physiological functions could help explain the absence of differences between CTRL and FIA fish at all sampling points, if an acute stress induced by the i.p. injection was in place. Nonetheless, irrespective of the role played by these opioids and their receptors, the present results clearly point out their importance during the earlier sampling points, as *muor*, *kor1* and *nopr* gene expression was strikingly lower at 7 days compared to 24 h post-injection, and was still lower at 21 days. Accordingly, carp head-kidney opioid receptors were observed to be upregulated in the first sampling points after i.p. injection with zymosan (6 and 24 h post-injection) but were gradually downregulated [19].

An exception was observed in *nopr*, the expression of which was upregulated at 14 days, only to recede to lower levels at 21 days. Specialized nociceptors, such as NOPR, detect noxious stimuli which by definition can or do cause tissue damage [66] and are usually associated to the inflammatory response and to later phases of this response [67]. Nociception in fish is linked to opioids and their receptors [66]. From the opioid receptors analyzed in the present study, it can be hypothesized that the upregulation of *nopr* mRNA levels can be related to ongoing chronic inflammation at 14 days post-injection, as already perceived by other parameters mentioned above.

**Figure 9:**
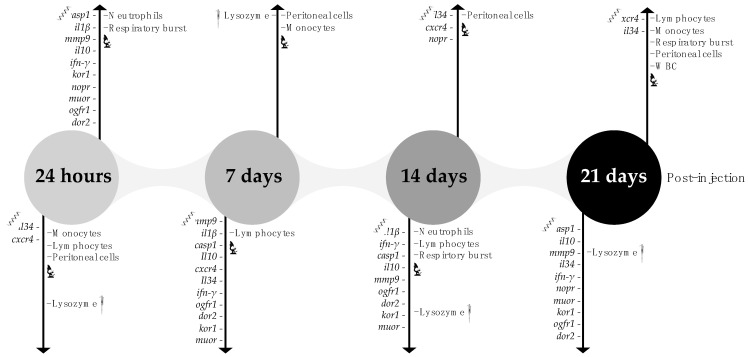
Timeline diagram of hypothetical mechanisms involved during a chronic inflammation on the peritoneal cavity and their potential markers. The diagram shows a qualitative assessment of the relative abundance of each parameter in inflamed fish over time. WBC—white blood cells, IgMp, plasma immunoglobulins M; *casp1*, caspase 1; *il1β*, interleukin 1 β; *mmp9*, matrix-metalloproteinase 9; *il10*, interleukin 10; *ifn-γ*, interferon γ; *cxcr4*, chemokine CXC receptor 4; *il34*, interleukin 34; *kor1*, κ-type opioid receptor-like 1; *nopr*, nociceptin opioid receptor; *muor*, μ opioid receptor; *ogfr1*, opioid growth factor receptor-like protein 1-like and *dor2*, δ-opioid receptor.

Considering the present results, a timeline diagram of hypothetical mechanisms involved during a chronic inflammation on the peritoneal cavity and their potential markers is illustrated on Figure 9.

## 5. Conclusions

In conclusion, and having in mind the possible presence of a stress-induced effect (i.p. air exposure and injection) that seemed to have masked part of the evaluated immune parameters, the results from the present study illustrate an orchestrated response to a peripheral and local immune stimulation. Immune defenses such as peripheral neutrophil populations, plasma proteases and lysozyme levels, as well as head-kidney mRNA expression of *il1β* and *casp1* genes were observed to change with inflammation but not in a particularly chronic way.

**Figure 10:**
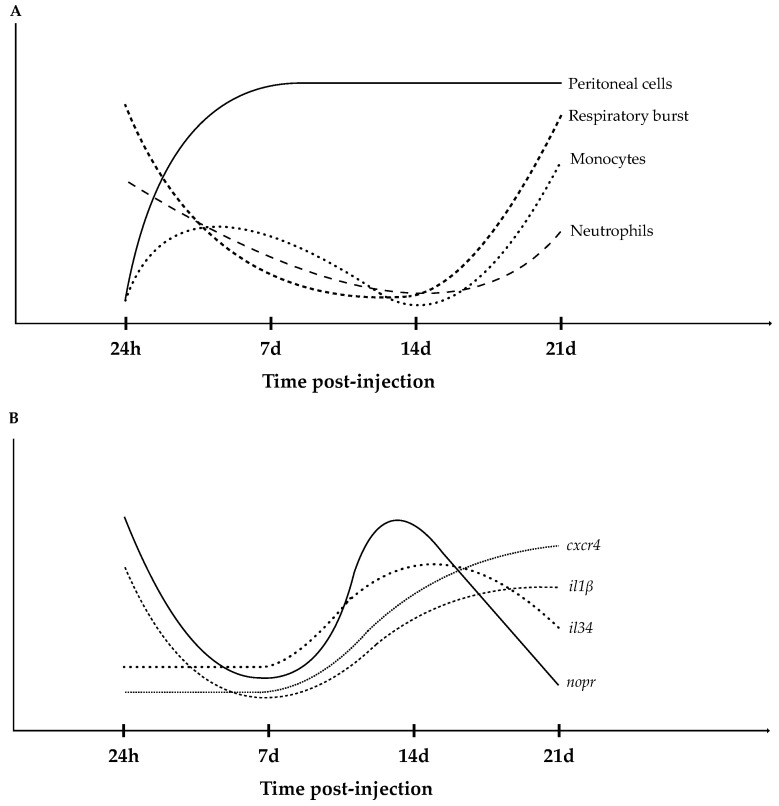
Schematic representation of main hematological (**A**) and gene expression (**B**) results from the present study. *cxcr4*, chemokine CXC receptor 4; *il34*, interleukin 34; *il1β*, interleukin 1 β and *nopr*, nociceptin opioid receptor.

Locally, inflammation was characterized by an intense recruitment of immune cells that was still evident 21 days after injection thus illustrating the chronic character of the immune response. Cellular response was also noticed peripherally with leukocyte numbers rising in the blood of FIA-injected fish, accompanied by an intensification of respiratory burst, suggesting that these cells were still actively fighting the phlogistic agent after 3 weeks (Figure 10A). Regarding the head-kidney molecular markers, *cxcr4* and *il34*, whose pathways have high relevance in chronic inflammation settings, stood out as relevant markers of chronic inflammatory responses (Figure 10B). The present study offers new data on the dynamics of a chronic inflammation context, and might serve as a baseline for the evaluation of immune responses in the European seabass.

## Figures and Tables

**Table 1:** Specifications of real-time qPCR assays including forward (F) and reverse (R) primers, GenBank ID (NCBI), efficiencies (Eff) of qPCR reactions and annealing temperature (Ta).

Gene	Acronym	GenBank ID	Eff (%) ^1^	Ta (°C)	Primer Sequence (5′–3′)
Elongation factor 1-α	*ef1α*	AJ866727.1	96.45	57	F: AACTTCAACGCCCAGGTCAT R: CTTCTTGCCAGAACGACGGT
40s ribosomal protein	*40s*	HE978789.1	92.96	55	F: TGATTGTGACAGACCCTCGTG R: CACAGAGCAATGGTGGGGAT
ribosomal protein L13 mRNA	*L13α*	DQ836931.1	127.31	55	F: AGTCCGGTGTCCCACTATCA R: GAGTTCCTCCAGGGTGAAGC
Interleukin 34	*il34*	DLAgn_00164750 ^2^	120.94	60	F: GGAAATACGCTTCAGGGATG R: GGCACTCTGTCGGGTTCTT
C-Reactive protein	*ptx*	EU660933.1	114.76	60	F: TGAAGTTTTTGCTGCTGGTG R: GGTTTCTTGTGGGAAGGTGA
Chemokine CXC receptor 4	*cxcr4*	FN687464.1	93.43	60	F: ACCAGACCTTGTGTTTGCCA R: ATGAAGCCCACCAGGATGTG
Interleukin 1 β	*il1β*	AJ269472.1	96.7	57	F: AGCGACATGGTGCGATTTCT R: CTCCTCTGCTGTGCTGATGT
Interleukin 10	*il10*	AM268529.1	116	55	F: ACCCCGTTCGCTTGCCA R: CATCTGGTGACATCACTC
Transforming growth factor-β	*tgf β*	AM421619.1	105.56	55	F: ACCTACATCTGGAACGCTGA R: TGTTGCCTGCCCACATAGTAG
Caspase 1	*casp1*	DQ198377.1	124	55	F: GTGTTTCAGATGCGGGAGGA R: ATTTAAGTTAACTCACCGGGGG
Interferon γ	*ifn-γ*	FQ310507.3	118.38	55	F: GTACAGACAGGCGTCCAAAGCATCA R: CAAACAGGGCAGCCGTCTCATCAA
Inmunoglobulin M	*IgM*	FN908858	91.64	60	F: AGGACAGGACTGCTGCTGTT R: CACCTGCTGTCTGCTGTTGT
Matrix-metalloproteinase 9	*mmp9*	FN908863.1	98.44	57	F: TGTGCCACCACAGACAACTT R: TTCCATCTCCACGTCCCTCA
μ opioid receptor	*muor*	DLAgn_00015310 ^2^	99.81	60	F: GTCACCAGCACCCTACCATT R: CGAGGAGAGAATCCAGTTGC
κ-type opioid receptor-like 1	*kor1*	DLAgn_00007470 ^2^	89.71	60	F: TCTGGTGCTTGTGGTAGTCG R: TGGCAGTCTCTGTGTCCTTG
Nociceptin opioid receptor	*nopr*	DLAgn_00125610 ^2^	97.57	60	F: CTCCTTTCTCATCCCTGTGG R: GTTGCGGTCCTTTTCCTTG
δ-opioid receptor	*dor2*	DLAgn_00062690 ^2^	108.06	60	F: CGCTTCTCGGTCTCCATAACT R: GGTCTCATTACTACTTGAAG
Opioid growth factor receptor-like protein 1-like	*ogfr1*	DLAgn_00128530 ^2^	96.79	60	F: GTTGGGAATGGAGATGGAAA R: GCTTCAGATTTTGGCTCAGG

^1^ Efficiency of qPCR reactions (represented in percentage) were calculated from serial dilutions of tissue RT reactions in the validation procedure. ^2^ Sequences obtained from databases dicLab v1.0c seabass genome.

## Data Availability

All data is provided in the main text or Supplementary Files.

## Supplementary Materials

The following supporting information can be downloaded at: https://www.mdpi.com/article/10.3390/biology11050764/s1, Table S1: Absolute values of total white, red and peritoneal cells, peripheral leukocytes (neutrophils, monocytes, lymphocytes and thrombocytes) and respiratory burst of European seabass 24 h, 7, 14 and 21 days after inflammation; Table S2: Plasma innate immune response parameters of European seabass 24 h, 7, 14 and 21 days after inflammation; Table S3: Quantitative expression of immune-related gene and opioid receptors of head-kidney of European seabass 24 h, 7, 14 and 21 days after inflammation; Table S4: Principal component analysis (PCA) eigenvalues; Table S5: Principal component analysis (PCA) eigenvectors.
