# Supplementary material for: Chronic Inflammation Modulates Opioid Receptor Gene Expression and Triggers Respiratory Burst in a Teleost Model
Source: Biology (Basel). 2022 May 17;11(5):764. doi: 10.3390/biology11050764 (PMC9138576; doi:10.3390/biology11050764)
Supplement: Supplementary file 1 [file biology-11-00764-s001.zip › biology-1682783-supplementary.pdf]

Supplementary File

Chronic inflammation modulates opioid receptor gene expression and triggers respiratory burst in a teleost model

Diogo Peixoto [1,2,3]. Marina Machado [2]. Rita Azeredo [2] and Benjamín Costas [2*]

[1] ICBAS - Instituto de Ciências Biomédicas Abel Salazar. Universidade do Porto. Porto. Portugal; dpeixoto@ciimar.up.pt (D.P.)

[2] CIIMAR - Centro Interdisciplinar de Investigação Marinha e Ambiental. Matosinhos. Portugal; mcasimiro@ciimar.up.pt (M.M.). mleme@ciimar.up.pt (R.A.) and bcostas@ciimar.up.pt (B.C.)

[3] Departamento de Biología. Facultad de Ciencias del Mar y Ambientales. Instituto Universitario de Investigación Marina (INMAR). CEIMAR-Universidad de Cádiz. Cádiz. Spain

* Correspondence: mleme@ciimar.up.pt (R.A.) and bcostas@ciimar.up.pt (B.C.)

Table S1 - Absolute values of total white, red and peritoneal cells, peripheral leucocytes (neutrophils, monocytes, lymphocytes and thrombocytes) and respiratory burst of European seabass after 24 hours, 7, 14 and 21 days after inflammation.

Parameters	Treatment	Time post-injection							
		24 hours		7 days		14 days		21 days	
WBC (x10^{4} µL^{-1})	CTRL	4.33 ± 0.93		5.83 ± 1.55		4.77 ± 1.45		9.95 ± 2.51	
	FIA	5.5 ± 1.05		4.8 ± 0.71		4.53 ± 1.43		10.25 ± 1.75	
RBC (x10^{4} µL-1)	CTRL	16.15 ± 4.27		13.35 ± 2.5		15.13 ± 5.79		18.63 ± 6.31	
	FIA	14.53 ± 3.27		17.05 ± 2.62		11.08 ± 4.49		17.53 ± 2.58	
Hematocrit (%)	CTRL	26.33 ± 3.83		31.17 ± 2.48		25.33 ± 3.67		25.2 ± 4.82	
	FIA	30 ± 4.86		30.33 ± 2.81		26.83 ± 1.72		28.6 ± 4.04	
Neutrophils (x10^{4} µL^{-1})	CTRL	0.11 ± 0.04	a#	0.04 ± 0.01	a#	0.07 ± 0.01	a	0.27 ± 0.09	b
	FIA	0.25 ± 0.04	b*	0.2 ± 0.06	ab*	0.1 ± 0.04	a	0.18 ± 0.05	ab
Monocytes (x10^{4} µL-1)	CTRL	0.08 ± 0.05	a	0.08 ± 0.01	a#	0.11 ± 0.02	ab	0.27 ± 0.09	b#
	FIA	0.16 ± 0.08	a	0.33 ± 0.1	b*	0.6 ± 0.09	ab	0.57 ± 0.2	c*
Lymphocytes (x10^{4} µL^{-1})	CTRL	1.22 ± 0.16	a#	1.85 ± 0.24	a	1.45 ± 0.36	a	1.69 ± 0.24	b
	FIA	1.96 ± 0.12	a*	1.54 ± 0.2	a	1.69 ± 0.24	a	4.29 ± 0.58	b
Thrombocytes (x10^{4} µL^{-1})	CTRL	2.95 ± 0.51		3.54 ± 0.69		3.59 ± 0.76		5.33 ± 0.76	
	FIA	3.19 ± 0.29		2.61 ± 0.38		2.6 ± 0.91		4.88 ± 0.27	
Total peritoneal cells (x10^{4} µL^{-1})	CTRL	5.83 ± 3.44		9.88 ± 7.31		6.47 ± 6.66		7 ± 5.97	
	FIA	11.7 ± 3.58	a	42.15 ± 23.15	b*	46.92 ± 9.8	b*	35.23 ± 8.73	b*
Respiratory burst (nmol O^{-}_{2})	CTRL	1277.56 ± 1121.38		375.28 ± 172.56		486.33 ± 388.98		743.67 ± 903.51	#
	FIA	2721.22 ± 1313.3	b	462.56 ± 175.24	a	800.39 ± 802.77	a	4134.39 ± 1294.93	b*

Two-way ANOVA

Parameters	Time	Treatment	Time *vs* Treatment	Treatment		Time			
				CTRL	FIA	24 hours	7 days	14 days	21 days
**WBC (x10^4 µL^{-1})**	<0.01	ns	ns	-	-	a	a	a	b
**RBC (x10^4 µL^{-1})**	ns	ns	ns	-	-	-	-	-	-
**Hematocrit (%)**	<0.01	ns	ns	-	-	ab	b	a	ab
**Neutrophils (x10^4 µL^{-1})**	<0.01	<0.01	<0.01	*	#	a	ab	b	c
**Monocytes (x10^4 µL^{-1})**	<0.01	<0.01	0.005	*	#	a	b	ab	c
**Lymphocytes (x10^4 µL^{-1})**	<0.01	0.003	0.002	*	#	a	a	a	b
**Thrombocytes (x10^4 µL^{-1})**	<0.01	0.015	0.137	#	*	a	a	a	b
**Total peritoneal cells (x10^4 µL^{-1})**	0.008	<0.01	0.018	*	#	a	b	b	ab
**Respiratory burst (nmol O^-_2)**	<0.01	<0.01	0.004	*	#	b	a	a	b

Values are presented as means ± SD (n = 6). Different low case letters stand for statistically significant differences attributed to sampling time. Symbols stand for significant differences between treatments. (Two-way ANOVA; Tukey post-hoc test; $p \leq 0.05$).

Table S2 - Plasma innate immune response parameters of European seabass after 24 hours, 7, 14 and 21 days after inflammation.

**Parameters**	**Treatment**	**Time post-injection**											
		**24 hours**			**7 days**			**14 days**			**21 days**		
**IgMp (OD 450 nm)**	**CTRL**	0.16	±	0.111	0.132	±	0.031	0.089	±	0.028	0.219	±	0.082
	**FIA**	0.161	±	0.056	0.109	±	0.027	0.084	±	0.036	0.255	±	0.099
**Inihibited trypsin (%)**	**CTRL**	81.193	±	0.297	81.617	±	0.992	83.682	±	1.641	82.612	±	2.78
	**FIA**	81.267	±	0.323	82.282	±	1.782	81.877	±	1.442	82.115	±	1.83
**Total protease (%)**	**CTRL**	7.755	±	1.364	10.291	±	0.808	9.08	±	1.035	9.98	±	0.92
	**FIA**	9.608	±	1.771	9.831	±	1.443	10.98	±	1.959	11.217	±	0.952
**Peroxidase (U perox)**	**CTRL**	38.693	±	19.154	39.48	±	20.309	60.283	±	17.627	37.911	±	21.77
	**FIA**	32.361	±	9.087	54.827	±	13.395	42.333	±	18.419	31.633	±	23.743
**Lysozyme (mg mL^{-1})**	**CTRL**	1.73	±	1.213	4.328	±	3.772	4.161	±	1.356	2.713	±	1.252
	**FIA**	2.372	±	1.493	9.721	±	3.949	5.008	±	1.204	4.459	±	1.772

Table S2 - Continuation.

Two-way ANOVA

Parameters	Time	Treatment	Time *vs* Treatment	Treatment		Time			
				CTRL	FIA	24 hours	7 days	14 days	21 days
**IgMp (OD 450 nm)**	<0.001	ns	ns	-	-	ab	a	a	b
**Inihibited trypsin (%)**	ns	ns	ns	-	-	-	-	-	-
**Total protease (%)**	0.003	0.006	ns	*	#	a	b	ab	b
**Peroxidase (U perox)**	ns	ns	ns	-	-	-	-	-	-
**Lysozyme (mg mL^{-1})**	<0.001	0.003	ns	*	#	a	b	ab	a

Values are presented as means ± SD (n = 6). Different low case letters stand for statistically significant differences attributed to sampling time. Symbols stand for significant differences between treatments. (Two-way ANOVA; Tukey post-hoc test; $p \leq 0.05$).

Table S3 - Quantitative expression of immune-related gene and opioids receptors of head-kidney of European seabass after 24 hours, 7, 14 and 21 days after inflammation.

Parameters		Treatment	Time post-injection			
			24 hours	7 days	14 days	21 days
Normalized mRNA expression	*tgfβ*	CTRL	1.466 ± 0.433	0.729 ± 0.426	1.175 ± 0.6	1.083 ± 0.382
		FIA	1.335 ± 0.415	0.881 ± 0.304	1.886 ± 1.058	1.035 ± 0.494
	cxcr4	CTRL	1.093 ± 0.591	1.046 ± 0.339	1.82 ± 1.357	1.148 ± 0.240
		FIA	0.65 ± 0.222	1.272 ± 0.382	2.389 ± 0.353	1.788 ± 0.548
	il1β	CTRL	3.002 ± 2.952	0.26 ± 0.211	1.007 ± 0.429	1.43 ± 0.618
		FIA	3.164 ± 2.214	0.931 ± 0.938	2.38 ± 1.417	1.399 ± 0.606
	mmp9[1]	CTRL	2.501 ± 0.85 [b]	0.528 ± 0.31 [a]	0.928 ± 0.43 [a]	1.439 ± 0.69 [ab]
		FIA	2.39 ± 0.67 [b]	0.852 ± 0.5 [a]	2.431 ± 3.07 [ab]	0.794 ± 0.34 [a]
	casp1	CTRL	5.321 ± 6.749	0.809 ± 0.456	1.754 ± 1.697	0.728 ± 0.610
		FIA	6.72 ± 4.398	1.287 ± 1.123	1.574 ± 0.923	1.349 ± 1.061
	il10	CTRL	6.336 ± 5.885	0.242 ± 0.163	1.075 ± 0.967	0.244 ± 0.159
		FIA	3.963 ± 1.977	0.453 ± 0.424	0.549 ± 0.436	0.623 ± 0.597
	il34	CTRL	0.752 ± 0.239	1.004 ± 0.957	1.333 ± 0.522	0.954 ± 0.41
		FIA	0.936 ± 1.045	1.854 ± 1.454	3.762 ± 2.65	1.667 ± 0.569
	hep	CTRL	19.787 ± 28.925	0.589 ± 0.663	4.242 ± 7.094	0.630 ± 0.802
		FIA	20.095 ± 17.693	1.742 ± 1.846	3.723 ± 5.56	2.917 ± 3.775
	ifn-γ	CTRL	14.162 ± 16.663	0.745 ± 0.963	3.398 ± 4.858	0.644 ± 0.82
		FIA	10.396 ± 6.699	1.163 ± 1.223	2.688 ± 4.029	1.082 ± 1.173
	**IgM**[1]	CTRL	0.937 ± 0.479	0.684 ± 0.611	0.858 ± 0.36	1.524 ± 0.064

	**FIA**	3.71 ± 2.796	1.142 ± 1.167	3.052 ± 3.006	1.316 ± 0.276

Table S3 - Continuation.

	ptx	**CTRL**	1.604 ± 0.573	1.029 ± 1.012	1.083 ± 0.695	1.546 ± 0.921
		**FIA**	1.397 ± 0.203	1.092 ± 0.463	1.073 ± 0.705	1.376 ± 0.469
	**Opioids receptors**					
**Normalized mRNA expression**	*nopr*	**CTRL**	3.551 ± 3.116	0.291 ± 0.313	2.001 ± 2.01	0.539 ± 0.309
		**FIA**	6.219 ± 4.819	0.315 ± 0.176	6.1 ± 7.654	0.301 ± 0.43
	muor	**CTRL**	11.026 ± 9.459	0.583 ± 0.741	1.452 ± 1.575	1.625 ± 1.557
		**FIA**	6.258 ± 4.457	1.837 ± 2.033	4.666 ± 5.547	2.462 ± 1.354
	kor1	**CTRL**	11.781 ± 9.359	0.506 ± 0.414	1.323 ± 1.819	0.388 ± 0.477
		**FIA**	7.621 ± 4.403	1.275 ± 1.239	1.296 ± 1.4	1.244 ± 1.005
	dor2	**CTRL**	9.674 ± 10.706	0.708 ± 0.79	2.98 ± 4.781	0.576 ± 0.596
		**FIA**	12.729 ± 10.237	1.821 ± 2.068	1.283 ± 2.062	1.448 ± 1.424
	ogfr1	**CTRL**	5.910 ± 8.871	0.555 ± 0.769	1.706 ± 1.669	0.853 ± 0.423
		**FIA**	5.839 ± 5.666	1.067 ± 1.191	1.4 ± 2.012	0.864 ± 0.779

[1] *non-parametric tests with Kruskal-Wallis pairwise comparisons*

Table S3 - Continuation.

**Two-way ANOVA**				**Treatment**		**Time**			
**Parameters**	**Time**	**Treatment**	**Time *vs* Treatment**	**CTRL**	**FIA**	**24 hours**	**7 days**	**14 days**	**21 days**
tgfβ	0.006	ns	ns	-	-	b	a	b	a
cxcr4	0.002	ns	ns	-	-	a	a	b	b
il1β	<0.001	0.05	ns	#	*	b	a	b	b
casp1	0.036	0.023	ns	#	*	b	a	a	a
il10	<0.001	ns	ns	-	-	a	b	b	b
il34	0.036	ns	ns	-	-	a	ab	b	ab
hep	0.02	ns	ns	-	-	b	a	ab	ab
ifn-y	ns	ns	ns	-	-	-	-	-	-
ptx	ns	ns	ns	-	-	-	-	-	-
**Opioid receptors**									
nopr	<0.001	ns	ns	-	-	b	a	b	a
muor	0.002	ns	ns	-	-	b	a	a	ab
kor1	<0.001	ns	ns	-	-	b	a	a	a

dor2	0.019	ns	ns	-	-	b	ab	ab	a
ogfr1	ns	ns	ns	-	-	-	-	-	-

Values are presented as means ± SD (n = 6). Different low case letters stand for statistically significant differences attributed to sampling time. Symbols stand for significant differences between treatments. (Two-way ANOVA; Tukey post-hoc test; $p \leq 0.05$).

Table S4 – Principal Component Analysis (PCA) eigenvalues.

	F1	F2	F3	F4	F5	F6	F7
**Eigenvalue**	10.316	4.064	2.400	1.691	1.241	0.885	0.403
**Variability (%)**	49.126	19.352	11.426	8.054	5.910	4.215	1.917
**Cumulative %**	49.126	68.478	79.904	87.958	93.868	98.083	100.000

Table S5 - Principal Component Analysis (PCA) eigenvectors.

	F1	F2	F3	F4	F5
**Plasma IgM**	0.002	-0.041	-0.542	-0.392	0.129
**Anti-protease**	0.016	0.122	-0.241	0.556	0.435
**Protease**	-0.201	0.294	-0.171	0.086	0.014
**Peroxidase**	-0.029	-0.223	0.441	-0.171	0.373
**Lysozyme**	-0.157	0.030	0.331	-0.146	0.464
**Igs**	-0.168	-0.347	-0.073	0.131	-0.324
tgfb	0.229	0.288	0.146	-0.012	-0.206
cxcr4	-0.140	0.383	0.213	-0.033	-0.032
il1b	0.253	0.272	-0.033	-0.121	0.063
mmp9	0.287	0.070	-0.017	-0.118	-0.149
casp1	0.276	-0.055	0.046	0.182	0.027
il10	0.291	-0.114	0.092	0.009	-0.056
igm	0.174	0.331	-0.212	0.096	0.063
ifn-y	0.280	-0.140	0.055	0.216	0.061
il34	-0.113	0.406	0.267	-0.015	0.038
ptx	0.230	0.067	-0.211	-0.398	0.211
nopr	0.253	0.105	0.167	0.262	-0.245
muor	0.277	0.112	0.081	-0.260	-0.073

kor1	0.267	-0.119	0.165	-0.102	-0.079
dor2	0.252	-0.255	0.017	0.019	0.180
ogfr1	0.276	-0.020	-0.071	0.196	0.322
